# Benefits of Cultural Activities on People With Cognitive Impairment: A Systematic Review

**DOI:** 10.3389/fpsyg.2021.762392

**Published:** 2021-11-25

**Authors:** Laia Delfa-Lobato, Joan Guàrdia-Olmos, Maria Feliu-Torruella

**Affiliations:** ^1^Faculty of Geography and History, University of Barcelona, Barcelona, Spain; ^2^Department of Social Psychology and Quantitative Psychology, Faculty of Psychology, University of Barcelona, Barcelona, Spain; ^3^Institute of Neuroscience, University of Barcelona, Barcelona, Spain; ^4^UB Institute of Complex Systems, University of Barcelona, Barcelona, Spain; ^5^Department of Applied Didactics, Faculty of Education, University of Barcelona, Barcelona, Spain; ^6^Institute of Research in Education (IRE), University of Barcelona, Barcelona, Spain

**Keywords:** systematic review, cognitive impairment, cultural activities, Alzheimer’s disease, dementia, arts and health, art therapy

## Abstract

Museums and cultural institutions are increasingly striving to respond to the interests and needs of the society that hosts them. This means, apart from other actions, that these institutions must be involved in the health and wellbeing of society, and the creation of cultural activities aimed at people with cognitive impairment, a group of individuals that is growing worldwide due to the aging of society and the increasing prevalence of dementia. The involved sectors are aware of the potential and benefits of activities for this population, even though there is much research to be conducted. To date, no systematic review has focused on the benefits of cultural activities for cognitively impaired people. This study aimed to explore the benefits of different modalities of cultural activities with evidence from 145 studies from various databases, which met the inclusion criteria. Significant improvements in general cognition, quality of life (QoL), emotional wellbeing, socialization, and communication were generally reported after interventions, with a reduction in depression symptoms. There was not enough evidence to prove memory, language, or daily functioning improvements attributable to cultural interventions. There were no significant reductions reported in apathy, sadness, agitation, or anxiety.

## Introduction

In recent years, museums, cultural institutions, and the heritage sector, in general, have increasingly undergone substantial changes, seeking to remove the image of stigmatized or intimidating spaces to become spaces where individuals can find wellbeing and quality of life (QoL) (Camic and Chatterjee, 2013). This change of concept appears when these institutions become aware of the interests, and especially the needs, of the community that hosts them (Jung, 2011; Desmarais et al., 2018).

Museums are proving to be a powerful ally in health and wellbeing programs, responding to a global trend in which they are becoming aware of everything that is likely to contribute something positive to the health and wellbeing of the population (Camic and Chatterjee, 2013; Chatterjee and Camic, 2015).

Reliable proof of this involvement is the growing number of museums and institutions that have decided to create and carry out programs to benefit people affected by diseases, such as dementia of Alzheimer’s type (DAT) and cancer, among others (Camic and Chatterjee, 2013; Chatterjee and Camic, 2015; Thomson et al., 2018).

In recent years, the heritage and cultural sector have been of interest to researchers and governments considering this population in terms of public health and wellbeing (O’Neill, 2010; Cuypers et al., 2012). Nevertheless, there is still a lack of scientific evidence that solidly proves the effectiveness of cultural practices in terms of health and wellbeing (Ander et al., 2011).

Within the wide range of public health and wellbeing issues, there is one that, due to its scope and implications, deserves special attention and not only from the cultural and heritage sector. These are people living with cognitive impairment (CI), a growing group of individuals affected by dementia and other conditions (O’Brien et al., 2003).

Cognitive impairment is a broad medical issue, as it is a condition that presents concomitantly with various diseases (Health Direct, 2020). The U.S. Agency for Disease Control and Prevention defines CI as memory loss, difficulty remembering, concentrating, or learning new things, as well as a feeling of confusion that gets worse or reiterates more frequently, even affecting decision-making and activities of daily life. In a mild stage, the person with CI may continue to lead a normal life, despite noticing changes in some of the cognitive functions mentioned above. In more severe stages, people with CI may see their decision-making or assessment of situations and circumstances diminished, as well as actions such as writing or speaking, making them become dependent on others (U.S. Department of Health and Human Services Centers for Disease Control and Prevention, 2011). Contrary to appearances, CI is not always a permanent and chronic condition; CI can present different levels of impairment, as it can be mild, intermediate, or severe (Hugo and Ganguli, 2014; Health Direct, 2020).

The causes of CI are very diverse and can be responsible for temporary or irreversible CI (Jurado et al., 2013; Bernardo and Carvalho, 2020). Some of the causes of reversible CI may include mental illnesses, infectious diseases, vitamin deficiency, dehydration, toxins, or medication reactions. The main causes of irreversible CI are traumatic brain injury, Parkinson’s disease (PD), Friedreich ataxia, Huntington’s disease (HD), amyotrophic lateral sclerosis (ALS), cardiovascular accidents, multiple sclerosis, normal aging, and neurodegenerative diseases. Some of these causes do not always have to be associated with CI (Jurado et al., 2013; Health Direct, 2020). It is important to highlight dementias, which are the most common cause of CI, responsible for 60–70% of cases worldwide and the third most common disease worldwide (World Health Organization, 2021). DAT (O’Brien et al., 2003), which is the most common cause of dementia, is incurable, as there are other conditions and diseases responsible for CI (Jurado et al., 2013). The second leading cause of CI, vascular dementia, accounts for up to 30% of cases (O’Brien et al., 2003).

Another important fact is to consider the estimated numbers of people that experts predict will be diagnosed with DAT in the coming years. The WHO estimates that the current number of people with dementia to be 50 million, and that number is expected to reach 152 million by 2050 (World Health Organization, 2021).

In addition to those with DAT, there are many family members and friends affected by this disease. According to the Pasqual Maragall Foundation, 54% of the population is now directly in contact with individuals who have DAT (Fundació Pasqual Maragall, 2021).

In this context and due to the lack of systematic reviews (SRs) on this subject, the aim of this investigation was to locate and analyze studies based on cultural activities to evaluate their benefits for individuals with CI who participated, independent of the cause of their conditions. To conduct the review, PRISMA guidelines (Moher et al., 2009; Hutton et al., 2016) were followed.

## Methods

### Study Eligibility

The inclusion criteria for this SR included (1) published empirical studies about arts and cultural as health and wellbeing activities for people diagnosed with initial- or middle-stage CI; (2) studies in English, French, Spanish, or Catalan; (3) studies published between 2010 and 2020 to analyze the newest resources, considering the previous studies outdated; (4) studies centered on interventions adjusted for groups or delivered individually; (5) with active or passive engagement of the participants in art and cultural activities (i.e., the creation of something artistic or hearing, seeing, or touching artistic/cultural elements); (6) cultural activities taking place in a museum or with cultural equipment or not; (7) interventions oriented to CI diagnosed people, CI diagnosed people and their caregivers, and/or CI diagnosed people and their family members; (8) quantitative or qualitative study designs; and (9) that it was not necessary to include pre-tests and post-tests measures, and reports and could or not include a control group.

**Population:** people diagnosed with CI.

**Intervention:** cultural and art-based interventions corresponding to UNESCO’s culture definition and framed within the cultural industries model defined by UNESCO (1982) and Throsby (2008).

**Comparators:** not required. Both studies with control and without control groups were included, as well as studies with and without pre-test and post-test measures and reports.

**Outcome:** any measure or description of intervention results.

**Study type:** interventional clinical trials (ICTs), SR, meta-analysis (MA), or SR and MA.

Reports were excluded if (1) they were defined as dissertations, books, book chapters, or non-empirical studies; (2) studies were cultural activities that did not correspond to those suitable by UNESCO’s culture definition (UNESCO, 1982) and framed within the cultural industries model defined by Throsby (2008).

### Search Strategy

To identify suitable papers for this review, a search was conducted in the databases Web of Science (WOS), SCOPUS, PubMed, and Medline. The search was restricted to 2010–2020.

The search was conducted in August 2020. Key search terms used were (muse* OR art OR “heritage site” OR “cultural engagement”). These key words were used in combination with the terms concerning CI (Alzheimer OR “CI” OR dement* OR “cognitive disfunction” OR “cognitive decline” OR “mild cognitive impairment”), using proximity Boolean search operators such as NEAR or W/n (*n* = 100) instead of AND when the database permitted it. To refine the search, artistic and cultural activity terms were included, as well as those concepts related to arts and health or art therapy as follows: (“arts and wellbeing” OR “arts and humanities” OR “arts and health” OR “reminiscence therapy” OR “art therapy” OR “dance therapy” OR “music therapy” OR “singing” OR “performing art” OR “theater” OR “cinema” OR “life story” OR “life review” OR “storytelling” OR “visual art” OR “creative art” OR “paint” OR “painting” OR “drawing” OR “collage” OR “pottery” OR “sculpture” OR “contemporary art” OR “art gallery” OR “photography”). For WOS and SCOPUS, the search was applied in all databases. A search procedure was built within the Medline database using Medical Subject Headings (MeSH), the PICO (patient/population, intervention, comparison/control and outcomes) strategy was followed (Fernández-Altuna et al., 2016) to optimize and adapt the search question, and the search strategy was developed and used in the rest of the databases.

### Data Extraction

Initially, data were extracted by one reviewer (LDL) using a data extraction form precisely designed by the three authors (LDL, JGO, and MFT) to cover these SR objectives. Later, a second reviewer (JGO) checked the data extraction to ensure accuracy. To settle the discrepancies, they were discussed between the three authors (LDL, JGO, and MFT). The data extraction form included the following information:

**General:** first author, publication year, and method.

**Participants:** Type of CI: Alzheimer’s, Parkinson’s, dementias, HD, mild CI, Creutzfeldt-Jakob disease, aging, and SR and MA. A “some CI types” option was included. Information about the age or mean age of participants; participation of “other participants,” such as family members, close friends, or informal caregivers was also included.

**Intervention:** individual or group format, number of participants, total duration, location, and time of day. Modality of intervention: music, performance arts, visual art, ceramics, storytelling, dance, literary arts, mixed, and in SR and MA. A “some modalities” option was included.

**Outcomes:** Cognition improved cognition generally, improved memory, and improved language. Motor function improved motor function generally. QoL and wellbeing generally improved QoL and improved emotional wellbeing and self-esteem. Behavioral symptoms reduced agitation and improved social interaction and communication. Psychological symptoms reduced depression, reduced anxiety, reduced apathy, and reduced sadness. Activities of daily living improved activities of daily living.

## Search Outcome

**FIGURE 1:**
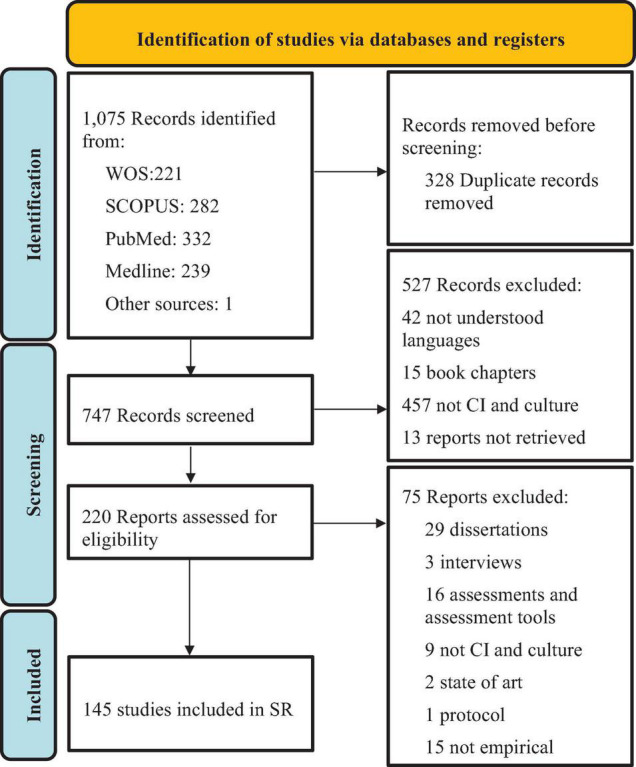
PRISMA flow chart of included studies.

A total of 1,075 documents were retrieved in the search, of which 328 were duplicates. A total of 747 records were screened by title and abstract, and 527 were removed because they did not meet the inclusion criteria. A total of 220 articles were full text screened, of which 145 studies met the inclusion criteria and were enrolled in the study (Supplementary Table 1). Among the 145 included studies, 30 were MA, SR, or SR and MA (20%), and 115 were ICT (80%). Of these, 38 were quantitative papers (26.20%), 42 were qualitative papers (28.96%), 17 were mixed-method papers (11.72%), 13 were comparative papers (both quantitative and qualitative) (8.97%), and 5 were papers based on individual interventions (3.45%) (Figure 1).

## Results

### Participants

#### Cognitive Impairment Typologies

Most of the studies focused on people living with dementia, in general, including 68 studies. It can seem obvious as dementias overall include DAT and Lewy body dementia, meaning that this typology is the most general one found in this SR, being a generic group that involves different CI typologies.

Dementias overall is followed by DAT and PD, with 22 and 21 selected studies, respectively. These are two of the most common CI typologies, as a large number of people worldwide have these conditions. The prevalence of these conditions makes them more visible than other CI typologies, and therefore, more initiatives are centered on DAT or PD.

It is always important to emphasize that aging is also considered a CI cause, so we considered it as a CI typology. In this study, 14 articles were focused on elderly individuals.

Mild cognitive impairment (MCI), which is very often a precursor to dementia, is another CI typology in the literature, with 11 studies focused on it in this study.

Almost trivially, two and one selected studies were focused on people with a diagnosis of HD and Creutzfeldt-Jakob disease, respectively. These two typologies of CI are less known among the general population.

#### Age of the Participants

When talking about the age of the intervention participants, it is important to specify that in this SR, ages have been classified as the commonly defined large age ranges (Urbanas et al., 2005).

None of the included studies worked with a group of participants whose mean age was under 60 years old. This means that all the studies were focused on older people.

### Participant Characteristics

#### Group Size

Some included studies (Argyle and Kelly, 2015; Sauer et al., 2016; Kontos et al., 2017; Ford et al., 2018; Deygout, 2019; Fields et al., 2019; Schneider et al., 2019; Wyatt and Liggett, 2019) mentioned the group size as an important aspect to consider. Most of these articles also indicate the benefits of person-centered approaches, something that can be related to the obtained results described as follows.

Most of the studies (58) included interventions in groups of fewer than 20 people, including 28 groups between 6 and 10 people. Of these, 21 reported QoL and wellbeing-related outcomes. Additionally, 15 included studies that conducted an intervention with groups of 6–10 people that reported improvements in communication and socialization. These results can be related to the benefits of a person-centered approach, as the center of the intervention is the person itself, with his or her history, interests, rights, values, and beliefs. In addition, these interventions, as always, treated participants with dignity and respect and tried to maximize their potential by offering them opportunities to learn new things, to enjoy new moments, and to share these experiences with new people (Edvardsson et al., 2008). However, these benefits are only possible when the professionals who are performing the intervention can dedicate enough time to each participant. Thus, this is probably the reason that small intervention groups report better outcomes related to person-centered approaches.

#### Location and Time of Day

In most of the included studies, the implications of performing the intervention in one specific place or another, as well as the time of day when it was carried out were not examined, so results are inconclusive.

Nevertheless, one study that compared the results of an object handling intervention concluded that the intervention can have the same results regardless of where it takes place (Camic et al., 2019). These findings suggest that the benefits of cultural activities are not related to the location where they take place, as assessed in the referenced article. In this case, the intervention took place in a museum and in a daycare center, obtaining positive and similar results related to emotional wellbeing at both locations (Camic et al., 2019). Likewise, the time of day when the interventions take place, distinguishing between morning and afternoon, does not seem to affect results, as this was the conclusion of a 4-month study based on art therapy (Deygout, 2019).

#### Other Participants—Informal Caregivers

Some interventions were especially thoughtful and offered to both people with CI and their caregivers. This is important, as very often the person who takes on the caregiver role is a close family member or, in some cases, a close friend of the impaired person (Wancata et al., 2005; Brodaty and Donkin, 2009; Carey et al., 2016). This makes this type of intervention especially meaningful, as it can be beneficial not only for the patient but also for his or her caregiver. It is important to understand that lives of these people turn around the disease, and they no longer have the quality time together they used to have. This type of intervention can offer them the opportunity to enjoy time together again and to remember how pleasant it was (Camic et al., 2014; Mittelman and Papayannopoulou, 2018).

Moreover, interventions that involve not only CI people but also their caregivers can improve QoL for both of them (Mondro et al., 2020). It is important to expose that, very often, the person who adopts the informal caregiver role must face multiple obstacles, such as financial problems or the difficulty of balancing caregiving with a social life and a career (Brodaty and Donkin, 2009). Consequently, this can result in some physical and mental illnesses, such as depression, anxiety, and other mental health issues, cardiovascular problems, or lower immunity (Wancata et al., 2005; Brodaty and Donkin, 2009). This makes it even more important to provide interventions that include caregivers, as sometimes they seem to be the forgotten ones. One of the most important benefits these interventions afford the caregivers is the possibility to share their experiences with others in the same situation (Mondro et al., 2020), making them feel understood and less alone, as social isolation is one of the negative consequences caregivers experience. Additionally, these interventions sometimes caregivers techniques they can use in their daily lives, making their burden as caregivers more manageable (Mondro et al., 2020).

### Modalities of the Interventions

#### Music

Notably, 28 studies reported music therapy as a modality in their investigations. Some of them included music therapy, as well as other modalities. There were mixed results regarding the type of CI interventions.

The most registered outcomes delivered by music therapy were improvements in general cognition, QoL, emotional wellbeing, or self-esteem and reductions in depression signs. It is important to add that although none of the ICTs included reported it, significant verbal and language fluency improvements were reported in three SRs (Schwartz et al., 2019; Fong et al., 2020; Lam et al., 2020).

Moreover, aphasia rarely appears in people with a diagnosis of CI due to the condition they are living with, as it affects some specific brain regions (Damasio, 1992). Nevertheless, music can be not only a therapeutic activity for this specific collective of CI people but also a way to communicate (Baird et al., 2017; Baird and Thompson, 2019). It has been documented that music cognition skills are preserved longer than language skills in people suffering from aphasia (Baird and Samson, 2015; Baird and Thompson, 2019), so music can be the perfect non-verbal form through which these people can communicate with their loved ones (Baird and Thompson, 2019). Additionally, in a specific case study, music brought to an informal caregiver of a person with DAT the opportunity to dance, and this provided them with physical contact and affection, something that sometimes is hard to find (Baird and Thompson, 2019). Nevertheless, even if the person with CI is not affected by aphasia, music or music combined with dance or theater can be a good way for people with CI to express themselves to others or share some memories with them (Dassa and Harel, 2019).

#### Performance Arts

Theater, as performing art, can be a useful tool to explore the deepest emotional, affective, and social parts of the human being (Fields et al., 2019). This type of intervention can provide socialization and communication as well as other outcomes related to emotional wellbeing. In this SR, seven studies mentioned performance arts.

Two types of intervention modalities should be distinguished: those in which people diagnosed with CI take an active role and those where they take a passive role. Among the selected articles, two specific ICTs (Kontos et al., 2017; Dassa and Harel, 2019) conducted interventions in which people with CI took an active role, and in both cases, the affective and emotional modes of expression and communication reached positive outcomes. Two other ICTs (Fields et al., 2019; Loewy et al., 2020) combined the passive and active roles of participants. In this case, it is interesting to note that in one intervention, caregivers were also involved (Loewy et al., 2020), and the emotional and affective relationship between them and those they cared for was reinforced.

#### Visual Arts

Both art appreciation and art making were the most performed interventions, with 74 people diagnosed with CI in the included studies. One of the reasons could be that art has always been a good way to communicate and express oneself; ancient philosophers already linked it with the life of the author and its way to express and communicate it (Wikström, 2005), which is sometimes harder for individuals with CI.

Two articles (Johnston and Terp, 2015; Couture et al., 2020) mention this type of therapy and what it offers to the person with CI and caregivers, often their partners. It must be assumed that when a partner in a couple is diagnosed with CI, it can be frustrating and disappointing for both partners, as their roles will change, communication will become more difficult, and shared pleasant moments will be harder to find (Johnston and Terp, 2015; Couture et al., 2020). Art can offer them a way to have quality time together and to share their thoughts and emotions (Couture et al., 2020) or create something together (García Cano, 2018; Mondro et al., 2020). The activity can help them regain a social life as they spend time with other people in a similar situation, making them feel understood and even finishing the intervention acting like a group of friends or becoming friends (García Cano, 2018). Additionally, visual arts, for individuals with CI, can be a powerful tool that they can use to express themselves, and it can help them fight against the idea of being a homogenous population, as they can share and tell their own stories through visual art and be understood as a communication facilitator (Peisah et al., 2011; Tucknott-Cohen and Ehresman, 2016; Hicks et al., 2019). The arguments above are directly linked with the obtained results, with facilitating socialization and communication being the most reported outcome. Furthermore, art therapy modality also reported outcomes such as general cognitive and memory improvements. Both QoL and emotional wellbeing were also reported as great outcomes, and some articles referred to a decrease in depression.

#### Ceramics

Two ICTs centered their interventions on pottery activities (Gontard et al., 2017; Pérez-Sáez et al., 2018). Both studies reported improvements in emotional wellbeing and self-esteem. It can be speculated that creating something artistic, as the participants of these two interventions did, could work well as a therapeutic activity focused on improving wellbeing and self-esteem in individuals with CI, as they can feel proud of themselves after being able to create something autonomously and after taking part in a significant activity (Pérez-Sáez et al., 2018).

#### Dance

Notably, 44 studies included dance therapy or combined some dance therapy with other modalities. Mixed results were reported on the effects of dance therapy oriented to individuals with CI; nevertheless, almost 50% percent of these studies emphasized improvements in motor function.

Active dance activities are often used with people with CI to improve their motor function (Guzmán-García et al., 2016, 2017). Overall, people with PD (Hashimoto et al., 2015; Clifford et al., 2017; De Natale et al., 2017; Albani et al., 2019) live with a chronic degenerative movement disorder that causes problems with motor function, although some non-motor symptoms are also associated with PD (Aguiar et al., 2016; Holmes and Hackney, 2017). This SR includes 21 dance-based studies conducted with people who had been diagnosed with PD.

Tango is one of the most commonly used dance styles with people diagnosed with PD in the selected articles when the objective was to improve motor function and QoL (Volpe et al., 2013; Rios Romenets et al., 2015; Albani et al., 2019). This could be related to the discovery of Hackney and Earhart (2010), who found that Tango, as a highly improvisational style, works better than other dance genres, such as ballroom dancing (Guzmán-García et al., 2013b), in improving motor function in general, and specifically, gait and balance, and it helps to improve QoL in people with PD. One of the reasons is that participants must be focused on the present moment, and they are not planning the complicated movements they are making (Hackney and Earhart, 2010; Koch et al., 2016). Some other studies also reinforce Hackney and Earhart’s findings related to the improvisational nature of Tango and the greatest improvements in people diagnosed with PD who participate in this genre of dance therapy, in comparison with dance therapy conducted through other genres of dance (Koch et al., 2016; Wiedenhofer et al., 2017).

QoL and emotional wellbeing and self-esteem are important outcomes reached through dance therapy (Guzmán-García et al., 2013a). Cognitive outcomes were also evident and important. These results are probably related to the cognitive demands of learning, for example, a new cerography or even simple steps, and thus, they have beneficial effects on the cognitive function of people with CI (Thogersen-Ntoumani et al., 2017). Some studies also reported improvements in social interaction and communication and a reduction in depressive symptoms.

Even though most of the dance therapy articles address dance as an active activity, viewing someone dancing also has benefits for individuals with CI. Viewing someone dancing can have almost the same benefits as listening to the music this person is dancing to, and so it is. However, the level or intensity of the benefits reached by only listening to the music is improved by listening to the music accompanied by the images of someone dancing to it (Cross et al., 2012).

#### Literary Arts

Three studies cited literary arts as one of the modalities explored in their research, although none of them centered their investigations exclusively on this practice, leading to inconclusive results.

#### Storytelling

Nine articles referred to storytelling. In this case, the results were concise, and improved emotional wellbeing and self-esteem, as well as social interaction and communication, were the main outcomes. These outcomes are related to a fact described in four of the selected articles, where participants found the space to reminisce about their lives, something that provided them with joy and happiness. Additionally, storytelling helped them socialize with others as they shared memories, even if it required making an effort (Loizeau et al., 2015; Swinnen and De Medeiros, 2018; Vigliotti et al., 2019).

#### Mixed

Nine ICTs reported on more than one type of intervention modality. The most frequently reported outcomes were those related to cognitive function, as well as psychological symptom improvements.

As previously stated, sometimes what is important about the results is not only the effect but also its intensity. In this study, this type of intervention must be considered, as some combinations of modalities can intensify an outcome to be reached through the intervention (Cross et al., 2012). It is important to have clear objectives and degrees of achievement and to have both in mind when planning the intervention.

## Discussion

This SR suggests that cultural interventions can improve general cognition, QoL, emotional wellbeing, self-esteem, communication, and socialization of participants with CI, as well as alleviate depression symptoms. Less generally, a specific modality of cultural activity can also help by improving the motor function of participants. However, this study also reveals that cultural activities failed to improve some specific aspects of cognition, such as memory or verbal and language fluency and daily functioning, in addition to the general inability to reduce apathy, sadness, agitation, and anxiety.

The described benefits of culture-based interventions in improving general cognition can be based on the fact that this type of intervention works as a training program for the brains of participants, and it can reinforce their neuroplastic and cognitive flexibility (Young et al., 2015, 2016; Alain et al., 2019; Brown et al., 2020), as it is in line with previous reports on the potential benefits of cultural and art-based activities in improving cognition in older adults (Alain et al., 2019).

The ability of culture-based activities to improve the QoL, wellbeing, and self-esteem of people living with CI suggests that in general, for older people who have been retired and not working or studying for years, taking part in a project where they can learn or create things again evokes a sense of personal growth can increase their life satisfaction (Brown et al., 2020). For people diagnosed with CI in particular, the experience can make them feel capable again, which is a beneficial effect of cultural interventions (Beauchet and Launary, 2014; Camic et al., 2014, 2016; Loizeau et al., 2015; Mittelman and Papayannopoulou, 2018).

The observed gains in communication and socialization for participants in these cultural-based activities can be explained by two facts. The first is that when participating in group interventions, participants are with other people who are experiencing the same situation, so this can help them empathize and thus begin to socialize with the members of the intervention group. This, which may seem a natural human being behavior, can be a very important opportunity for people living with CI, as loneliness and a minimal and reduced social life are quite frequent among this population (Nikmat et al., 2015; Evans et al., 2018). The second reason is that many artistic modalities, such as plastic art and theater, can be useful communication tools to explore and express oneself (Wikström, 2005; Peisah et al., 2011; Tucknott-Cohen and Ehresman, 2016; Baird and Thompson, 2019; Fields et al., 2019; Hicks et al., 2019).

The findings that cultural-based activities help heal depression suggest that there is a scientific basis for the effectiveness of art-based interventions in reducing depression and other psychological symptoms (Stuckey and Nobel, 2010). Nevertheless, more clinical trial studies are needed to obtain well-founded conclusions.

Regarding the results linked to the improvements in motor function, it is important to say that most of the studies that reported this outcome was dance-based and was focused on people living with a disease such as PD, which causes, overall, problems with motor function (Hashimoto et al., 2015; Clifford et al., 2017; De Natale et al., 2017; Albani et al., 2019).

Moreover, dance style-based activities can also be important resources to adapt for ethnic and racial groups. For example, Salsa and Bachata are the two dance styles culturally appropriate to include in interventions for US Hispanic minorities involved in CI-oriented projects and may make these interventions more attractive for this population. It is an important consideration when trying to reduce disparities in the participation of minority populations (Aguiñaga and Marquez, 2017; Thogersen-Ntoumani et al., 2017). In folk recreations, which in this study were mixed modality interventions, it is also important to consider racial and ethnic groups in the design of activities or interventions. These interventions will likely be more attractive to them and can have important benefits by helping them recall memories (Li and Li, 2017).

While this study described the improvements that resulted from cultural-based activities that targeted individuals with CI, it is important to note that qualitative study results mainly support improvements related to social interaction and communication, QoL, and wellbeing, as these outcomes are usually measured through qualitative mechanisms. Quantitative study results are more oriented to outcomes such as improvements in motor function or cognitive and executive function, normally based on cognitive or motor empirical measures usually treated through a quantitative perspective.

However, although the outcomes of a study are important and must be considered and assessed as has been performed in this research, it is equally important to define and identify which outcomes are best and how they might be reached. The interventions should be conducted through one modality type or another. Additionally, it is important to know that the same outcomes can be reached through different modalities but at different intensities (Cross et al., 2012). As an example, listening to music in a passive way and listening to the same music with someone dancing to it have the same benefits; however, in the second intervention modality, the benefits were more intense. Specifically, in both interventions, the benefits are memory enhancement and relief of depression symptoms; however, when someone is dancing to music, relief of depressive symptoms improves significantly in comparison with listening to the music passively, and the effect lasts longer (Cross et al., 2012). Another example is comparing two modalities of intervention having nothing in common, for example, art therapy and music reminiscence. In this case, as participants are more active and involved in the art therapy intervention than in the music reminiscence intervention, the benefits obtained as a result of the first intervention are more significant (Mahendran et al., 2018). Therefore, it is important to plan the interventions and the outcomes and try to forecast not only the outcomes but also their intensities to design intervention and conduct it through the most appropriate modality. In addition, when planning the goals to be reached, it is important to take into consideration that interventions directed at achieving subjective goals, such as wellbeing, easily measure what participants want and truly enjoy, as they are not basing the success of the intervention on biomedically measurable goals. Therefore, this difference between the goals to be achieved must also be considered (Beard, 2012).

Another step that must be pointed out should be the final stage in every intervention, the evaluation. A well-performed evaluation must include a pre-test and post-test, and an assessment to verify if the observed effects caused by the interventions were retained by participants for some time after the intervention (Cross et al., 2012; Narme et al., 2012; De Natale et al., 2017; Lazarou et al., 2017; Zafar et al., 2017; Cheung et al., 2018; Zhao et al., 2018; Alain et al., 2019; D’Cunha et al., 2019). It will also be a powerful and empiric tool to truly prove that the benefits of cultural activities last beyond the intervention itself, which is something that adds value to this type of practice.

The studies included in this review were highly heterogeneous, which complicates the extrapolation of its findings. There were obvious variations between studies, such as intervention modality, CI typology, different size groups, and different age groups. However, this heterogeneity must be assumed to be normal since art and culture encompass a broad audience. Moreover, some other limitations of this SR must be mentioned. Most limitations come from the previously mentioned heterogeneity. For example, some studies did not distinguish between self-esteem and emotional wellbeing or socialization improvements and communication improvements, so the option was to unify the outcomes as most studies did. In addition, different scales of measure, as well as different criteria between qualitative and quantitative studies, were used, making exact comparisons difficult. Furthermore, many intervention assessments were based on caregivers, informal caregivers, or the same people with CI observations and feelings. Another limitation was that, in some studies, it was not clear if improvements or changes in participants were related to the intervention or the natural disease progression.

From the results and observations obtained and derived from this review, there are some recommendations and advice for future studies and interventions. It is important to unify the criterion used to assess cultural-based activities and interventions to facilitate the agglutination of information so that it can be more easily understood and examined. Another important observation is that cultural activities are important not only for the people living with CI but also for the family and friends who are taking care of them, as they also benefit from these interventions. Finally, as this SR suggests that cultural activities may provide some benefits to individuals with CI, it could be interesting to combine cultural activities more often with pharmacological therapies, and it will be interesting to extend this type of practice to other types of CI.

## Data Availability Statement

The original contributions presented in the study are included in the article/Supplementary Material, further inquiries can be directed to the corresponding author/s.

## Author Contributions

LD-L, JG-O, and MF-T contributed to the conception and design of this study. LD-L performed the search and the analysis and wrote the manuscript. All authors contributed to manuscript revision and read and approved the submitted version.

## Conflict of Interest

The authors declare that the research was conducted in the absence of any commercial or financial relationships that could be construed as a potential conflict of interest.

## Publisher’s Note

All claims expressed in this article are solely those of the authors and do not necessarily represent those of their affiliated organizations, or those of the publisher, the editors and the reviewers. Any product that may be evaluated in this article, or claim that may be made by its manufacturer, is not guaranteed or endorsed by the publisher.
