# Supplementary material for: Benefits of Cultural Activities on People With Cognitive Impairment: A Systematic Review
Source: Front Psychol. 2021 Nov 25;12:762392. doi: 10.3389/fpsyg.2021.762392 (PMC8656402; doi:10.3389/fpsyg.2021.762392)
Supplement: Supplementary file 1 [file Table_1.DOCX]

Supplementary Material

Table 1: Characteristics of included studies.

| **Reference** | **Number Subjects** | **Mean age intervention/control** | **Total duration** | **Intervention modality** | **Diagnosis** | **Method** | **Reported findings** | **Results** |
| --- | --- | --- | --- | --- | --- | --- | --- | --- |
| Baird2018 | 1 | 77/ND | ND | Music | Alzheimer | Qualitative interventional clinical trial | Preservation of music skills and use of it for communication. | ++ |
| Dedreu2012 | - | - | - | Music | Parkinson | Metanalysis | Significant improvement in walking velocity, gait and QoL. | ++ |
| Fang2017 | - | - | - | Music | Alzheimer | Systematic review | Music therapies can reduce cognitive and memory decline. | +++ |
| Lam2020 | - | - | - | Music | Dementia | Systematic review | Music therapies bring reductions in anxiety, depression and apathy and improvements in verbal fluency. | ++ |
| Mittelman2018 | 22 | 71-79/71-79 | 13 weeks | Music | Dementia | Mixed methodology (quantitative and qualitative) | Were improved QoL, social support and self-esteem. | +++ |
| Miyazaki2020 | 26 | 80/83,58 | 12 weeks | Music | Elderly | Quantitative interventional clinical trial | Demonstrated improvements in cognitive function. | +++ |
| Moreno-Morales2020 | - | - | - | Music | Dementia | Systematic review and metanalysis | Music could be a powerful treatment strategy. | ++ |
| Sanchez2016 | 22 | 88,73/88,09 | 16 weeks | Music | Dementia | Comparative interventional clinical trial with controlled trial | Improvements regarding agitations and depression symptoms. | +++ |
| Thomas2018 | - | - | - | Music | Dementia | Systematic review | Arts interventions seems to improve QoL. | + |
| Vink2012 | 77 | 82,42/81,76 | 16 weeks | Music | Dementia | Comparative interventional clinical trial with randomized controlled design | Decrease in agitated behaviors. | + |
| Vink2014 | 74 | ND/ND | 16 weeks | Music | Dementia | Comparative interventional clinical trial with randomized controlled design | Reduction of agitation, depression, and apathy symptoms. | ++ |
| Cheung2016 | 165 | 85,7 & 84,5/85,58 | 6 weeks | Music and dance | Dementia | Comparative quantitative interventional clinical trial | It enhanced cognitive functions, improved anxiety, and depressive symptoms. | + |
| Cross2012 | 50 | 76 & 77/ ND | 1 session | Music and dance | Elderly | Comparative quantitative interventional clinical trial | Dance group had lower depression scores than music group and improved in recognition memory. | ++ |
| Schwartz2019 | - | - | - | Music and Dance | Huntington disease | Systematic review | Results show positive impacts on language production and memory, behavior, QoL, Motor and cognitive functions, well-being, and reduction of anxiety symptoms. | +++ |
| Kontos2015 | 23 | 87,8/ND | 12 weeks | Performance arts | Dementia | Mixed methodology (quantitative and qualitative) | Enhance verbal and embodied communication. | ++ |
| Dassa2019 | 12 | 80/ND | 10 weeks | Performance arts and music | Dementia | Comparative qualitative interventional clinical trial | Expanded their emotional and creatives modes of expression. | ++ |
| Loewy2019 | 46 | ND/ND | 12 weeks | Performance arts and music | Dementia | Comparative qualitative interventional clinical trial | Enhanced QoL and provide opportunities for social connection. | +++ |
| Badia2017 | - | - | - | Visual arts | Dementia | Systematic review | Improvements in general cognition, social interaction, emotional well-being, and reduction of agitation symptoms. | ++ |
| Beard2011 | - | - | - | Visual arts | Dementia | Systematic review | Subjective well-being, help ensure that | ++ |
| Belver2017 | 12 | 84,4/ND | 8 weeks | Visual arts | Dementia | Qualitative interventional clinical trial | Improvements in well-being and social inclusion. | +++ |
| Beuachet2014 | 128 | 83,7/85,5 | 1 session | Visual arts | Dementia | Quantitative interventional clinical trial | Participants were less likely to die, and could improve posture, mobility and accelerate rehabilitation for daily living activities. | ++ |
| Brown2020 | 17 | 72/ND | 12 weeks | Visual arts | Elderly | Quantitative interventional clinical trial | Results show significant improvements in executive function, cognitive flexibility, and life satisfaction. | +++ |
| Camic2013 | 24 | 78,3/ND | 8 weeks | Visual arts | Dementia | Mixed methodology (quantitative and qualitative) | Interventions support personhood, stimulate cognitive processes of attention and concentration, and it fosters social inclusion. | ++ |
| Camic2015 | 12 | 58-94/ND | 8 weeks | Visual arts | Dementia | Qualitative interventional clinical trial | Intellectual stimulation, offering social inclusion opportunities and positive emotional and relational effects. | +++ |
| Camic2017 | 80 | 74,81/ND | 28 weeks | Visual arts | Dementia | Quantitative interventional clinical trial | Results showed positive increases in well-being. | +++ |
| Ciccarelli2019 | 25 | 77,33 & 75 &74,85/77,38 | 1 session | Visual arts | Parkinson | Quantitative interventional clinical trial | It can evoke emotional responses. | ++ |
| Couture2020 | 5 | 71,7/ND | ND | Visual arts | Alzheimer | Qualitative interventional clinical trial | Provides pleasure and helps expressing themselves. | +++ |
| D'cunha2019 | 25 | 84,6/ND | 6 weeks | Visual arts | Dementia | Quantitative interventional clinical trial | Post-interventions depressive symptoms decreased, and memory and verbal fluency improved. | ++ |
| Deshmukh2018 | - | - | - | Visual arts | Dementia | Systematic review | Art therapies may help to slow cognitive deterioration, improve QoL and it can have a social function and can affect emotional well-being. | + |
| Deygout2020 | 12 | 86/ND | 12 weeks | Visual arts | Alzheimer | Quantitative interventional clinical trial | Individual components of self-esteem improved satisfactory. | ++ |
| Dominguez-Toscano2017 | 25 | 60-80/60-80 | 12 weeks | Visual arts | Dementia | Mixed methodology (quantitative and qualitative) | Improvements in QoL, cognitive function, social inclusion, and depression symptoms. | ++ |
| Eekelaar2012 | 6 | 78/ND | 4 weeks | Visual arts | Dementia | Qualitative interventional clinical trial | Episodic memory was enhanced and improved participants mood and confidence and reduced isolation. | ++ |
| Fields2019 | 15 | +65/ND | 3 sessions | Visual arts | Elderly | Quantitative interventional clinical trial | Depression scores decreased after the intervention and improved psychological well-being. | ++ |
| Flatt2014 | 20 | ND/ND | 1 session | Visual arts | Alzheimer | Qualitative interventional clinical trial | Intervention that provide cognitive stimulation, social connections, and self-esteem of the participants. | ++ |
| Garcia-Cano2018 | 12 | ND/ND | 3 sessions | Visual arts | Alzheimer | Qualitative interventional clinical trial | Increment of confidence, well-being, and social interaction. | ++ |
| Graham2018 | ND | ND/ND | 4 weeks | Visual arts | Dementia | Qualitative interventional clinical trial | Reminiscence, and self-identity. | +++ |
| Gross2013 | 76 | 84,28/ND | 12 weeks | Visual arts | Dementia | Quantitative interventional clinical trial | Significant improvements on five well-being domains. | + |
| Guseva2019 | 1 | 85 | 8 weeks | Visual arts | Alzheimer | Qualitative interventional clinical trial | Positive changes in four domains of psychological well-being. | ++ |
| Hattori2011 | 39 | 75,3/73,3 | 12 weeks | Visual arts | Alzheimer | Quantitative interventional clinical trial | Improvements in the apathy scale and significant improvement in their QoL. | ++ |
| Hazzan2016 | 16 | 80/ND | 24 weeks | Visual arts | Dementia | Qualitative interventional clinical trial | Interventions improves social interaction and strength social connections. | +++ |
| Hicks2017 | 2 | ND/ND | 2 sessions | Visual arts | Dementia | Qualitative interventional clinical trial | They were encouraged to express their message. | +++ |
| Hsiao2020 | 54 | >80/<80 | 12 weeks | Visual arts | Dementia | Quantitative interventional clinical trial | Art therapy has positive effect on agitated and anxious behaviors. | +++ |
| Humphrey2017 | 8 | 80/ND | 27 sessions | Visual arts | Dementia | Qualitative interventional clinical trial | Art making and appreciation offer social benefits. | +++ |
| Ilali2018 | - | - | - | Visual arts | Elderly | Systematic review | Art interventions have positive impacts on various aspects of mental health such as depression, anxiety, agitation, QoL, self-esteem and social participation. | +++ |
| Ishihara2019 | 8 | 51-85/ND | 4 weeks | Visual arts | Alzheimer and mild cognitive impairment | Quantitative interventional clinical trial | Observed mood improvements and decrease of depression symptoms. | ++ |
| Jones2018 | 12 | 81,4/ND | 12 weeks | Visual arts | Dementia | Mixed methodology (quantitative and qualitative) | Well-being benefits can be materialized in economic terms. | +++ |
| Kirk2018 | 43 | 80,18/79,86 | 5 weeks | Visual arts | Alzheimer | Quantitative interventional clinical trial | Improvements in global cognitive function and semantic memory. | ++ |
| Lazar2016 | 9 | ND/ND | 18 sessions | Visual arts | Elderly | Qualitative interventional clinical trial | It manifests the importance of having models based on empathy and empowerment. | +++ |
| Lea2020 | ND | ND/ND | 96 weeks | Visual arts | Dementia | Qualitative interventional clinical trial | Helped them to socialize with their community. | +++ |
| Lee2018 | 68 | 71,1/71,1 | 36 weeks | Visual arts | Mild cognitive impairment | Qualitative interventional clinical trial | Results show significant improvements in memory and cognitive function. | +++ |
| Lesniewska2018 | 190 | 81,5/ND | From 1991 to 2012 | Visual arts | Alzheimer | Qualitative interventional clinical trial | It made them feel engaged with their group, making them feel proud of themselves while depression symptoms were reduced. | +++ |
| Loizeau2015 | 4 | 75,75/ND | 9 weeks | Visual arts | Dementia | Mixed methodology (quantitative and qualitative) | Positive change in subjective well-being having positive effects on their mood. | +++ |
| Lopez2015 | 16 | ND/ND | 20 weeks | Visual arts | Alzheimer | Qualitative interventional clinical trial | Improved their general cognition, their QoL, their security and self-esteem and their ability to socialize. | +++ |
| Luyten2017 | 35 | ND/ND | 8 sessions | Visual arts | Dementia | Qualitative interventional clinical trial | No cognitive/emotional responses were recorded or evaluated. | ++ |
| Masika2020 | 39 | 73,4/72 | 6 weeks | Visual arts | Mild cognitive impairment | Quantitative interventional clinical trial | Improvement in depressed mood scores, cognitive function, and psychological well-being. | ++ |
| Masika2020 | - | - | - | Visual arts | Elderly | Systematic review and metanalysis | Art therapy may improve global cognition, and reduced depression symptoms and anxiety. | +++ |
| Mondoro2018 | 6-des | ND/ND | 8 weeks | Visual arts | Dementia | Qualitative interventional clinical trial | It helps to improve their communication as well as their interaction with others, and their QoL. | +++ |
| Newman2018 | 48 | 84,9/Nd | 12 weeks | Visual arts | Dementia | Qualitative interventional clinical trial | Increased communication, improved self-esteem and had influenced positively their relationship with caregivers and family. | +++ |
| Peisah2011 | 1 | 82/ND | 6 sessions | Visual arts | Dementia | Individual case interventional clinical trial | Person living in dementia improved in well-being and reduced agitation. | +++ |
| Richards2018 | 52 | 74,8/74 | 8 weeks | Visual arts | Dementia | Mixed methodology (quantitative and qualitative) | Significant improvements in self-esteem and QoL of people living with dementia. | +++ |
| Roe2014 | 17 | 75-92/ND | 24 weeks | Visual arts | Elderly | Qualitative interventional clinical trial | Evidence of benefits on people’s health, well-being and QoL, and it also helped lift their mood and social engagement. | +++ |
| Sauer2014 | 38 | ND/ND | 60 weeks | Visual arts | Dementias | Mixed methodology (quantitative and qualitative) | Results indicate improvement in well-being and social interaction. | +++ |
| Savazzi2020 | 20 | 79,1/77,8 | 7 weeks | Visual arts | Alzheimer | Quantitative interventional clinical trial | Improvement in general cognition, amelioration in language, executive function and QoL. | +++ |
| Schneider2018 | 1 | ND/ND | 1 session | Visual arts | Dementia | Individual case interventional clinical trial | Promote self-esteem and social confidence in people living with dementia. | +++ |
| Seifert2017 | 6 | 23-85/23-85 | 13 weeks | Visual arts | Dementia | Quantitative interventional clinical trial | Remarkable improvements in self-esteem and physicality. | +++ |
| Shoesmith2020 | 15 | 84,4/ND |  | Visual arts | Dementia | Mixed methodology, quasi-experimental. | Enhanced social functioning and QoL. | +++ |
| Shrestha2016 | 1 | 42/ND | 24 weeks | Visual arts | Creutzfeldt-Jakob | Individual case interventional clinical trial | Patients and families can benefit from visual art therapies. | +++ |
| Stallings2010 | 3 | Late 70 – Early 80 | 2 sessions | Visual arts | Dementia | Qualitative interventional clinical trial | An opportunity to convey information they might not be capable to verbalize. | +++ |
| Tan2018 | 8 | +70/ND | 6 weeks | Visual arts | Alzheimer | Qualitative interventional clinical trial | Stimulating space for self-discovery and socializing. | +++ |
| Thomas2017 | 1 | ND/ND | ND | Visual arts | Alzheimer | Individual case interventional clinical trial | This type of therapies can be helpful for as to express emotional and psychological conflicts. | +++ |
| Thomson2017 | 115 | 65-94/ND | 10 weeks | Visual arts | Elderly | Mixed methodology (quantitative and qualitative) | Participants improve in psychological well-being. | +++ |
| Tietyen2017 | 8 | ND/ND | 8 weeks | Visual arts | Dementia | Quantitative interventional clinical trial | Indicated improvements in cognitive performance and QoL. | +++ |
| Tucknott-Cohen2016 | 1 | +85/ND | 17 weeks | Visual arts | Alzheimer | Individual case interventional clinical trial | Benefits of art therapy regarding communication, QoL and agitation. | +++ |
| Tyack2015 | 24 | 75 & 66/ND | 2 weeks | Visual arts | Dementia | Mixed methodology (quantitative and qualitative) | Findings suggest well-being benefits. | ++ |
| Ullán2012 | 21 | ND/ND | 5 – 10 sessions | Visual arts | Dementia | Qualitative interventional clinical trial | It gives satisfaction to participants during the creative process and the achieved results. | +++ |
| Windle2016 | 100 | ND/ND | 12 weeks | Visual arts | Dementia | Mixed methodology (quantitative and qualitative) | It is important to measure before, during and after the intervention’s participants QoL and well-being. | ++ |
| Windle2017 | 125 | 81,4/ND | 12 weeks | Visual arts | Dementia | Mixed methodology (quantitative and qualitative) | Participants experienced improvements in social connectedness, self-esteem and QoL, and sadness reduction. | ++ |
| Windle2017 | - | - | - | Visual arts | Dementia | Systematic review and Protocol | Absence of rigorous methodology to demonstrate art-based benefits. | +++ |
| Wyatt2018 | 8 | +65/ND | 8 sessions | Visual arts | Dementia | Qualitative interventional clinical trial | Through painting people living with dementia can communicate feelings. | +++ |
| Young2015 | 13 | 78,8 & 81,8/ND | 8 weeks | Visual arts | Dementia | Qualitative interventional clinical trial | Verbal fluency and lifetime memory were increased. | +++ |
| Young2015 | - | - | - | Visual arts | Dementia | Systematic review | Creative activities can have positive impact on cognitive function, in particular on attention, | +++ |
| Yu2020 | 49 | 71,14/71,44 | 12 weeks | Visual arts | Mild cognitive impairment | Quantitative interventional clinical trial | Gains in memory and cognitive function. | +++ |
| Zhao2018 | 93 | 70,6/69,5 | 16 weeks | Visual arts | Mild cognitive impairment | Quantitative interventional clinical trial | Improvements in general cognitive functions, memory, and language function. | +++ |
| Alain2019 | 53 | 67,7 & 68,9/68,5 | 12 weeks | Visual arts and music | Elderly | Comparative quantitative interventional clinical trial | Enhancement of auditory evoked responses, also gaining in response speed and improving in naming. | ++ |
| Lokon2016 | 67 | ND/ND | 4 weeks | Visual arts and music | Dementia | Comparative, observational, and quantitative interventional clinical trial | The impact of this intervention was overall in well-being domains as well as social interest. | + |
| Mahendran2017 | 90 | 60-85/60-85 | 36 weeks | Visual arts and music | Elderly | Comparative qualitative interventional clinical trial | Positive outcomes were measured regarding mood changes in anxiety and depression, and it helped to trigger cognitive responses. | ++ |
| Narme2012 | 33 | ND/ND | 4 weeks | Visual arts and music | Alzheimer | Comparative quantitative interventional clinical trial | Positive impact on the emotional state of the participants. | +++ |
| Pongan2017 | 59 | 78,8 & 80,2/ND | 12 weeks | Visual arts and music | Alzheimer | Comparative quantitative interventional clinical trial | Significant decrease of anxiety and depressive symptoms assessed. | ++ |
| Pongan2019 | 59 | 78,8 & 80,2/ND | 12 weeks | Visual arts and music | Alzheimer | Comparative quantitative interventional clinical trial | Provide benefits on the patients’ well-being. | ++ |
| Gontard2016 | 4 | 84,25/ND | 10 sessions | Ceramics | Alzheimer | Mixed methodology (quantitative and qualitative) | Improvements in self-esteem and helps in social interaction. | +++ |
| Perez-Saez2018 | 30 | 79,97/ND | 12 weeks | Ceramics | Dementia | Quantitative interventional clinical trial | Significant positive impact on mood and self-esteem. | +++ |
| Albani2019 | 10 | 63,1/ND | 5 weeks | Dance | Parkinson | Quantitative interventional clinical trial | Significant improvement on their QoL and improvements in mobility. | +++ |
| Arguiñaga2017 | 21 | 75,4/ND | 16 weeks | Dance | Mild cognitive impairment | Qualitative interventional clinical trial | Enjoyable and safe mode of physical activity, making them feel good. | ++ |
| Bognar2016 | 15 - 20 | ND | ND | Dance | Parkinson | Qualitative interventional clinical trial | Increase enjoyment in life, social interaction, QoL and helps in terms of motor abilities. | +++ |
| Clifford2017 | 7 | 69,6/ND | 12 weeks | Dance | Parkinson | Quantitative interventional clinical trial | Results suggest improvements in participants QoL and motor manifestation of the illness. | +++ |
| De Natale2016 | 16 | 66/70 | 10 weeks | Dance | Parkinson | Quantitative interventional clinical trial | Effectively impacts on motor and executive functions. | +++ |
| Guzman2016 | 10 | 81-95/ND | 8 weeks | Dance | Dementia | Quantitative interventional clinical trial | Beneficial in terms of mood and socialization. | + |
| Guzman2016 | 10 | 78-95/ND | 12 weeks | Dance | Dementia | Qualitative interventional clinical trial | Results show positive effects on social interaction, depression, anxiety, and agitation symptoms as well as self-esteem. | ++ |
| Guzman-Garcia2012 | 13 | 80,5/ND | 6 weeks | Dance | Dementia | Qualitative interventional clinical trial | Enhance positive emotional state, reducing levels of agitation and anxiety. Improving mobility and socialization and communication. | ++ |
| Guzman-Garcia2012 | - | - | - | Dance | Dementia | Systematic review | Problematic behaviors and agitation decreased, and social interaction and well-being improved. | ++ |
| Hashimoto2015 | 46 | 67,9 & 62,7/69,7 | 12 weeks | Dance | Parkinson | Quantitative interventional clinical trial | Significant improvements in motor function, cognitive function, and mental symptoms such as apathy and depression. | ++ |
| Hwang2015 | - | - | - | Dance | Elderly | Systematic review | Cognitive ability showed significant improvements, as well as flexibility and muscular strength. | +++ |
| Kalyani2019 | 33 | 65,24/66,50 | 12 weeks | Dance | Parkinson | Quantitative interventional clinical trial | Important improvements in selected cognitive skills and psychological symptoms. | ++ |
| Kalyani2019 | - | - | - | Dance | Parkinson | Systematic review and metanalysis | Dance may ameliorate general motor symptoms, particularly gait and global cognition. | ++ |
| Karkou2017 | - | - | - | Dance | Dementia | Systematic review | No studies were included, no results were obtained. | - |
| Kropacova2019 | 99 | 69,16/68,37 | 24 weeks | Dance | Elderly and mild cognitive impairment | Quantitative interventional clinical trial | Subtle improvements in executive functions, memory, and depressive symptoms. | + |
| Kunkel2017 | 14 | 72/ND | 10 weeks | Dance | Parkinson | Qualitative interventional clinical trial | Positive impact on participants enjoyment and well-being. | ++ |
| Lazarou2017 | 129 | 65,89/67,92 | 40 weeks | Dance | Mild cognitive impairment | Quantitative interventional clinical trial | Results show benefits in cognitive functions. | +++ |
| Lewis2014 | 37 | 65,94/64,5 | 10 weeks | Dance | Parkinson | Quantitative interventional clinical trial | Reduction in anger and depression symptoms as well as well-being. | +++ |
| Lotzke2015 | - | - | - | Dance | Parkinson | Systematic review and metanalysis | Dance interventions can have positive effects on motor function and QoL. | ++ |
| Lyons2018 | - | - | - | Dance | Dementia | Systematic review | Improvement of general health and well-being. | ++ |
| Marquez2017 | 57 | 64,8/66,4 | 16 weeks | Dance | Elderly | Quantitative interventional clinical trial | Participants showed grater improvement in episodic memory and global cognition. | +++ |
| Meng2019 | - | - | - | Dance | Elderly | Systematic review and metanalysis | Dance interventions can improve global cognition and in particular memory domain. | +++ |
| Michels2018 | 13 | 66,44/75,5 | 10 weeks | Dance | Parkinson | Quantitative interventional clinical trial | Greater improvements were in motor measures. | ++ |
| Ravelin2011 | 13 | 71-89/ND | ND | Dance | Dementia | Qualitative interventional clinical trial | It triggers processes related it different basic elements like other art forms. | ++ |
| Rios-Romenets2015 | 33 | 63,2/64,3 | 12 weeks | Dance | Parkinson | Quantitative interventional clinical trial | Participants demonstrated improvements in dynamic balance and some other motor function items, as well as cognitive function. | ++ |
| Rios-Romenets2017 | 33 | 63,2/64,3 | 12 weeks | Dance | Parkinson | Quantitative interventional clinical trial | Answer to a commentary on “Riosromenets2015”. | - |
| Shanahan2015 | - | - | - | Dance | Parkinson | Metanalysis | Interventions with beneficial effects for participants’ motor function. | ++ |
| Shanahan2017 | 90 | 69/69 | 10 weeks | Dance | Parkinson | Quantitative interventional clinical trial | Dancing may improve QoL. | ++ |
| Sharp2014 | - | - | - | Dance | Parkinson | Systematic review and metanalysis | Dance significantly can improve motor scores, gait speed and QoL. | +++ |
| Solla2019 | 20 | 67,8/67,1 | 12 pax | Dance | Parkinson | Quantitative interventional clinical trial | Significant improvements in motor and cognitive function, depression symptoms and apathy. | +++ |
| Thogersenntoumani2017 | 30 | 72,6/ND | ND | Dance | Elderly | Qualitative interventional clinical trial | Social dance to improve cognitive and social functioning. | +++ |
| Volpe2013 | 24 | 61,6/65 | 24 weeks | Dance | Parkinson | Quantitative interventional clinical trial | Enhance mobility, balance and QoL. | ++ |
| Westheimer2015 | 14 | 66,2/ND | 8 weeks | Dance | Parkinson | Mixed methodology (quantitative and qualitative) | Were reported physical, emotional, and social benefits. | ++ |
| Zafar2016 | 109 | 68,4 & 82,3/ND | 12 weeks | Dance | Parkinson and elderly | Quantitative interventional clinical trial | Participants improved their social life. | ++ |
| Zhang2019 | - | - | - | Dance | Parkinson | Systematic review and metanalysis | Dance therapy is beneficial in improving executive function and global cognitive function, depression, and apathy. | ++ |
| Abrahão2018 | 15 | ND/ND | 4 weeks | Storytelling | Dementia | Qualitative interventional clinical trial | Encourage participants in terms of expressivity and creativity and stimulate participants reminiscence. | +++ |
| Capstick2015 | 10 | 86,7/ND | 6 weeks | Storytelling | Alzheimer | Qualitative interventional clinical trial | Positive findings on well-being, social participation, and occupational diversity. | +++ |
| Chung2014 | 23 | ND/ND | 4 weeks | Storytelling | Dementia | Mixed methodology (quantitative and qualitative) | Media presentations alleviate agitation and anxiety and bring positive feelings. | ++ |
| Hydén2013 | 8 | ND/ND | 20 weeks | Storytelling | Dementia | Qualitative interventional clinical trial | Improvements in social interaction and communication. | +++ |
| Swinnen2017 | 8 | ND/ND | 10 weeks | Storytelling | Dementia | Qualitative interventional clinical trial | Decreased participants insecurities and became more inclined to speak freely. | +++ |
| Vigliotti2018 | 22 | 80-97/ND | 24 weeks | Storytelling | Dementia | Mixed methodology (quantitative and qualitative) | Benefits on participants’ QoL. | ++ |
| Chen2013 | 104 | 86,1/84,4 | 24 weeks | Mixed | Dementia | Quantitative interventional clinical trial | Interventions have a positive effect on behavioral and psychological symptoms, such as delusion, hallucination, and agitation. | +++ |
| Eades2016 | 6 | ND/ND | 3 – 4 weeks | Mixed | Dementia | Qualitative interventional clinical trial | Fight isolation and helps in terms of well-being and dementia visualization. | +++ |
| Ford2018 | ND | ND/ND | 24 weeks | Mixed | Elderly | Qualitative interventional clinical trial | Positive impacts reducing participants anxiety, agitation and improving their social interaction and communication skills. | +++ |
| Li2016 | 48 | 83,1/81,1 | 16 weeks | Mixed | Dementia | Quantitative interventional clinical trial | Increased significantly | +++ |
| Mahendran2018 | 68 | 71,1 & 71,6/70,6 | 36 weeks | Mixed | Mild cognitive impairment | Qualitative interventional clinical trial | Some improvements in depression and anxiety. | ++ |
| Skingley2020 | 16 | ND/ND | ND/ND | Mixed | Dementia | Qualitative interventional clinical trial | Engagement project for caregivers and people with dementia to restore their relationship. | +++ |
| Tischler2019 | 21 | ND/ND | 48 weeks | Mixed | Dementia | Qualitative interventional clinical trial | This model of inclusive intervention should be fully considered in dementia care as a useful tool to create a sense of community. | +++ |
| Bernardo2020 | - | - | - | More than one modality | Elderly | Systematic review | Cultural engagement could be a protective factor for cognitive function, it can make QoL and well-being improve and depression and agitation decrease. | +++ |
| Cowl2014 | - | - | - | More than one modality | Dementia | Systematic review | Identify challenges of this type of studies such as small sample sizes, short follow-up. | ++ |
| Curtis2018 | - | - | - | More than one modality | Elderly | Systematic review | Cultural interventions can be potentially beneficial at treating behavioral and emotional symptoms of dementia. | +++ |
| De Medeiros2014 | - | - | - | More than one modality | Dementia | Systematic review | Cultural arts can generate social and behavioral changes. | ++ |
| Fong2020 | - | - | - | More than one modality | Mild cognitive impairment | Systematic review | Art interventions report improvements in global cognition, language, and motor function. | +++ |
| Ward2020 | - | - | - | More than one modality | Dementia | Systematic review | Participatory arts activities can bring social, cognitive, and emotional benefits. | +++ |

+++ Mainly positive conclusions/results regarding cultural activities effectiveness.

++ Positive and negative conclusions/results in the same proportion regarding cultural activities effectiveness.

+ Overall negative conclusions/results regarding cultural activities effectiveness and some positive aspects.

- **N**o conclusions/results presented.
